# How do plant demographic and ecological traits combined with social dynamics and human traits affect woody plant selection for medicinal uses in Benin (West Africa)?

**DOI:** 10.1186/s13002-024-00655-2

**Published:** 2024-02-09

**Authors:** Carlos Cédric Ahoyo, Thierry Dèhouegnon Houéhanou, Alain Sèakpo Yaoitcha, Bénédicte Perpétue Akpi, Armand Natta, Marcel Romuald Benjamin Houinato

**Affiliations:** 1 Laboratory of Applied Ecology, Faculty of Agronomic Sciences, University of Abomey-Calavi, Cotonou, Benin; 2 Laboratory of Ecology, Botany and Plant Biology, Faculty of Agronomy, University of Parakou, Parakou, Benin; 3 Institut National des Recherches Agricoles du Bénin, Abomey-Calavi, Benin; 4 Laboratoire de Biomathématiques et d’Estimations Forestières, Faculté des Sciences Agronomiques, Université d’Abomey-Calavi, Cotonou, Benin

**Keywords:** Ethnobotanical interview, Vegetation survey, Plant use value hypothesis, Extinction of wild populations

## Abstract

**Background:**

Several hypotheses have been used in ethnobotany to explain the plant’s selection criteria by people for their daily needs. Thus, it is important to assess synergy and complementarity among them, especially, those concerning the plant use value, social dynamics and human traits. The study aims to (i) highlight people’s socio-economic factors, and plant ecological traits that affect the plant use-availability dynamic (PUD); and (ii) assess the available species diversity effect on ethno-medicinal knowledge diversity in Benin.

**Methods:**

Ethnobotanical interviews were carried out to quantify the importance of local species in different ecological zones of Benin with 590 traditional medicine actors. Vegetation surveys were done to assess species availability within 337 plots of 50 m x 40 m or 60 m x 30 m, depending on the climatic zone, for a total of 61.6 ha, established in 15 forests distributed within the 10 phytodistricts of Benin. The plant use availability hypothesis was quantified as a dynamic link between species use value and availability (PUD). A general and mixed linear models were used to assess the significance of each factor’s effect on PUD. Pearson correlation test was applied on Shannon diversity index considering inventoried species in the field and those which were cited by people, for the available species diversity effect on ethno-medicinal knowledge diversity assessment.

**Results:**

A hundred and twenty woody medicinal plants, mostly trees (68.33%), were sampled. Growth form and its interaction with phytodistrict have a significant effect (*p*: 0.005) on PUD. The less available trees were the most used in the phytodistricts 3, 4, 8 and 10. PUD varies significantly according to social factors (*p*: 0.007). Ethnicity, age and main activity were the most quoted social factors which influenced the PUD. Ethnicity and age have various effects considering the phytodistricts. Moreover, the influence of age changes following the main activity. Plant selection did not solely link to the surrounding diversity (*r*: − 0.293; *p*: 0.403). Within some phytodistricts, especially those of 3, 4, 8 and 10, the less available tree species were the most requested.

**Conclusion:**

It is urgent to reforest vegetation patches in some phytodistricts (3, 4, 8 and 10) of Benin with widely requested and no available species to avoid the extinction of their wild populations. This concerns *Cassia sieberiana* DC., *Anonychium africanum* (Guill. & Perr.) C. E.Hughes & G. P. Lewis, *Pterocarpus erinaceus* Poir., *Cola millenii* K. Schum., *Azadirachta indica* A. Juss., *Khaya senegalensis* (Desr.) A. Juss., *Pseudocedrela kotschyi (Schweinf.)* Harms, *Treculia africana* Decne. ex Trécul, *Uapaca heudelotii* Baill., *Vitellaria paradoxa* C. F. Gaertn., *Kigelia africana* (Lam.) Benth. and *Newbouldia laevis* (P. Beauv.) Seem. ex Bureau.

**Supplementary Information:**

The online version contains supplementary material available at 10.1186/s13002-024-00655-2.

## Background

Traditional ecological knowledge helps tribal communities adapt to socio-ecological changes, improving the long-term sustainability of their livelihood strategies and fostering social–ecological resilience [1]. Plants are fundamental elements for human life, considering their multiple functions for food, medicine, culture, agroforestry, and technology. Moreover, rural people strongly depend on woody species for their daily needs [2]. In developing countries, 75% of the population appeals to phytotheapy as primary healthcare source [3]. However, these medicinal plants are under increased pressure due to demography growth [4]. These resources’ protection for human well-being and biodiversity management depends on the people’s understanding of the plants’ selection criteria. In these last decades, several ecological hypotheses stimulated ethnobotanical studies development, aiming to explain plant resources use by local communities [5]. They include versatility, diversification, and availability hypotheses [6] which attempt to explain the increasing or disproportionately large number of exotic plants utilized in traditional medicine. They acknowledge traditional medicine as a dynamic system and suggest possible drivers of this phenomenon [6].

The availability hypothesis states that plants are used for medicine due to their greater accessibility or local abundance [7, 8]. Availability could be a physical distance from a home or community to the location where a plant grows in the wild, seasonality, abundance, price, as well as access to markets, gardens, or natural areas where plants are found [7, 9]. The behaviour of the resource use by local people is often ecologically driven, based on an abundance of resources [10]. In general, people tend to select the highly qualitative plants that could be the easiest to find. This mechanism seems similar to the ecological stoichiometry that studies the balance of energy and multiple chemical elements in ecological interactions [11].

The availability hypothesis seems to have been confounded for a long time to the ecological apparency hypothesis. Indeed, earlier tests of the ecological apparency hypothesis [12] were a simple prediction of plant usage by their availability. Most available plants tend to be more used by people, and thus, susceptible to being incorporated into the local culture. Increasing a given taxa abundance implied an increase in its local relative importance. People tend to use the easiest found plants [7]. However, the ecological apparency hypothesis assessment should consider also physiological and pharmacological features of plant species. This hypothesis was assessed with several research axes and methods. Among, the plethora of suggested indices, the use value has been proposed by some authors to assess the relative importance of a plant within a community. This method is based on the use number assigned to a plant by the whole informant group [13, 14], and requires a high number of interviews. Thus, the importance of a plant depends on its total use number. Accordingly, the seldom mentioned plants could be considered less valuable. Although, the availability hypothesis seems well discussed in ethnobotany [15–17], just a few studies assessed it through ecological processes [18–20]. In general, plant appearance is based on ecological factors, such as life forms. Indeed, trees seem more available, and thus more used, especially in traditional medicine, than shrubs and other smaller life forms [21]. There has been an established positive relationship between the structural parameters of plant community and their use intensity [7].

The ecological availability hypothesis assessment was based on a single predictor, plant availability effect on their selection. Whereas, the plant selection by people for use is based on several criteria (botanical family, life form, local abundance, etc.) simultaneously. Thereby, a multiple predictors approach seems more suitable to assess at the same time, multiple socio-demographical and ecological effects on the plant use-availability dynamic. This approach differs from the available ones by its ability to involve simultaneously several traits, related to the plant use value hypothesis.

Although the effects of sociocultural factors such as age, sex, ethnicity [22–24], profession, sensibility [25] and knowledge [26–28] on plant selection, were well established, it is important to highlight the ecological ones which drive this selection. Thus, for a given region, the whole factors which lead the plant selection, especially the woody medicinal species will be highlighted. Correspondingly, the threats that they faced should be better addressed by a proper selection of the socio-cultural or ecological factors whose effects could increase or reduce the threats.

Thereby, the paper through plant use value, and age, gender, and dynamics of knowledge hypothesis assessment, aims to (i) highlight people’s socio-economic factors, and plant ecological traits effects on plant use-availability dynamic; and (ii) assess the available species diversity effect on ethno-medicinal knowledge diversity.

## Methods

### Study area

**Fig. 1:**
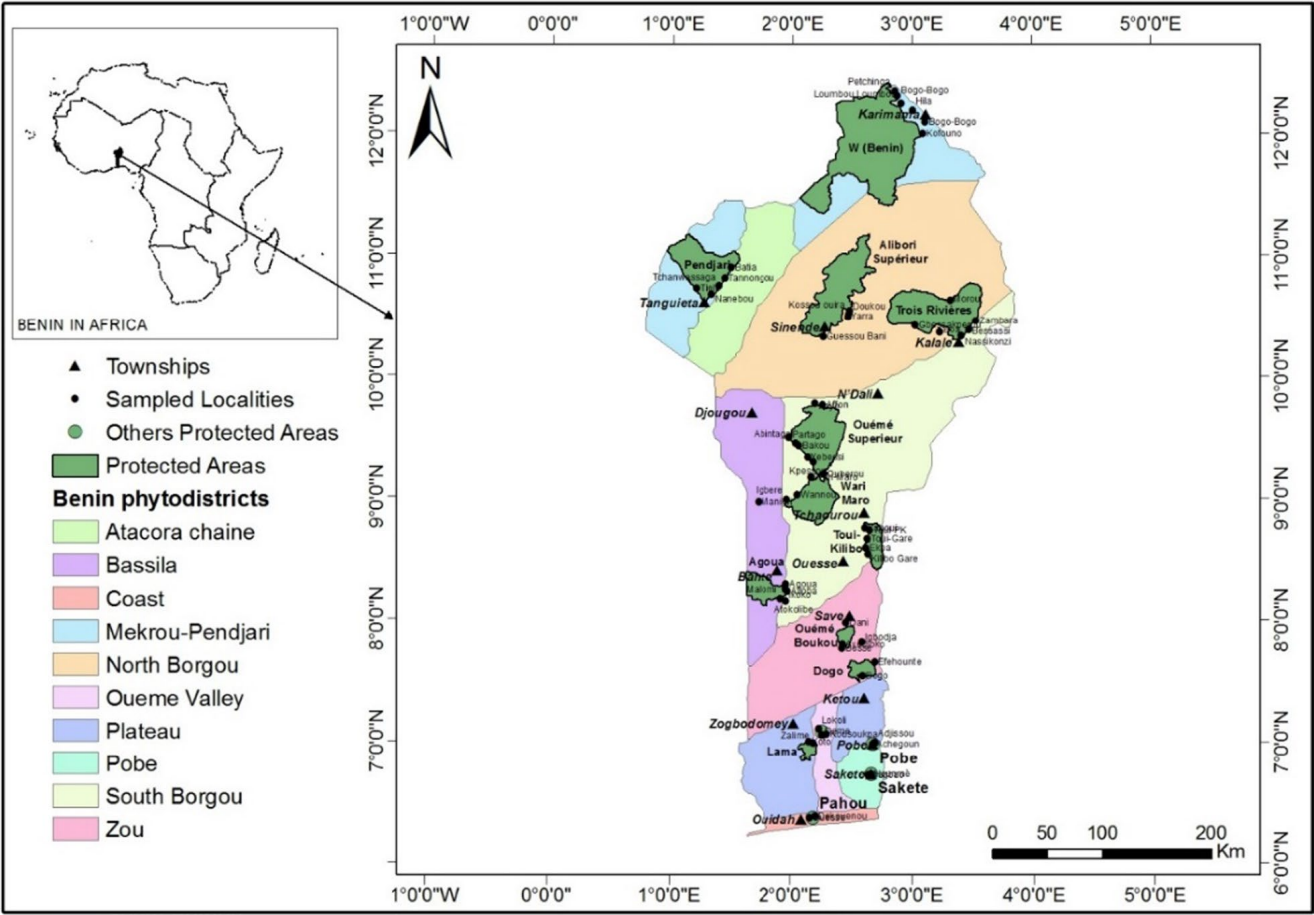
Study area situation showing sampled forests and localities

The study is realized in the forests of 10 phytodistricts of Benin obtained based on climate-flora homogeneity [29, 30] (Fig. 1) within the three climatic zones (Table 1). Benin is an occidental African country of 114,763 km^2^ for extent with a subequatorial climate [31]. It was characterized by geomorphological, geological, hydrographical, edaphic, climatic, and demographical diversities, which explain the fragmentation and the diversity of vegetation and their floristic composition variability [32].

**Table 1:** Characteristics of climatic zones in Benin

Climatic zones Characteristics	Guineo-Congolean	Sudano-Guinean	Sudanian
Longitude Latitude	1°–2° 45′ E 6° 30 ′–7° 30′ N	1°550–2°250 E 8°500–9°100 N	2°250 E 3°40′ E 9°100 N–12°30′ N
Climate	subequatorial	Sudanian humid	tropical arid (Sahelo-Soudanian)
Rainy season	bimodal: April-July and September–November	unimodal: April–October	unimodal: March–April to October–November
Dry season	bimodal: November-March and July–September	unimodal: November–March	unimodal: October–November to March–April
Rainfall	900 mm (west)–1300 mm (east)	900–1200 mm	800–1000 mm
Temperature	27 °C (average)	21–40 °C (average: 32 °C)	26–31.5 °C
Humidity	80%	65%	always under 65%

### Sampling and data collection

The ethnobotanical data including socio-economics factors (Table 2) were collected in 60 localities around 15 forests considering the 10 phytodistricts. In total, 590 traditional healers were interviewed. The informants’ selection followed their activity (professional healers, plant organ sellers, community elders) and proximity with plant formations (classified or not forests). In each sampled village, local authorities helped to identify the most relevant informants, after introducing and explaining the purpose of the visit and the aims of the survey.

**Table 2:** Informants sociodemographic characteristics

Factors	Number	Percentage (%)
Phytodistrict		
1	24	4
2	41	7
3	33	6
4	73	12
5	25	4
6	63	11
7	118	20
8	51	9
9	109	18
10	53	9
Sex		
Female	64	11
Male	526	89
Age		
Young (< 40)	112	19
Older ([40; 60[)	225	38
Elder (> 60)	253	43
Marital status		
Single	22	4
Monogamic	422	72
Polygamous	3	1
Divorced/widower	143	24
Household status		
Household chief	559	95
Conjoint	9	2
Grand parent	18	3
Child	4	1
Main activity		
Healer	313	53
Breeder	139	24
Medicinal plant sellers	131	22
Other	7	1
Instruction		
Illiterate	473	80
Primary	67	11
Secondary	50	8
Ethnicity		
Fon	66	11
Goun	4	1
Adja	5	1
Bariba	43	7
Dendi	12	2
Gourmache	16	3
Boo	7	1
Lokpa	7	1
Pila Pila	5	1
Wama	3	1
Biali	1	0
Yom	9	2
Mahi	25	4
Nago	114	19
Peuhl	161	27
Gando	1	0
Xoli	33	6
Ayizo	4	1
Tori	4	1
Yoruba	8	1
Ifè	52	9
Isha	10	2

Data concerning their sociodemography, the treated illnesses and the used woody species were thus collected for the plant use-availability dynamic testing. For the woody species, a distinction was made about the life form. Hence, trees were distinguished from shrubs. The information was borrowed from the literature review and confronted with the field data and the Analytical flora of Benin [32]. The height at adult stage was taken into account. The woody species whose height at adult stage is over 3 m was considered as “tree”, and “shrub”, if lesser.

The Code of Ethics of the International Society of Ethnobiology [33] was strictly followed, and the purpose of the study was explained before conducting the interviews. Verbal informant consent was obtained from the participants, who were assured of confidentiality and anonymity. A cordial relationship was established with the informants at the beginning of the study. The informants were interviewed after introducing and explaining the purpose of the visit and the aims of the survey. Written informed consent for participation was not required for this study, according to national legislation and institutional requirements.

In addition, ecological data concerning the species availability were recorded within the 15 forests (Fig. 1). In these forests, rectangular plots were set to measure the Diameter at Breast Height (DBH) for adult individuals for stability of data requirement. The quadratic diameter was computed for multistemmed individuals. The plots size was 2000 m^2^ (50 m × 40 m) within Guineo-Congolese (phytodistricts 1, 2, 3 and 4) and 1800 m^2^ (60 m × 30 m) for Guineo-Sudanian (phytodistricts 5, 6 and 7), and Sudanian ones (phytodistricts 8, 9 et 10). In total, 337 plots were established for 61.6 ha of sampled forests.

### Data analysis

The collected data on people’s knowledge of plant use in traditional medicine allows the calculation of the medicinal use value of the cited species. The Use Value (UV) was computed to assess the importance of each species through phytodistricts, and life forms. It followed Philips and Gentry [12], simplified by Rossato et al. [34] formulae:

$${\text{UV}}=\sum \frac{{\text{Ui}}}{{\mathrm{n}}}$$

With *Ui*, the use number mentioned by an informant *i*, and *n*, the total number of informants who mentioned the species.

The ecological availability of useful tree species has been assessed including their relative frequency, relative density and relative dominance. Importance value index (IVI) was calculated for each woody species (DBH ≥ 10 cm) according to the following formula:

$${\text{IVI}}=\sum ({\text{Fr}}+{\text{Dr}}+{\text{Gr}})$$

with Fr = relative frequency, Dr = relative density and Gr = relative dominance.

IVI is a quantitative index, which varies from 0% (absence of dominance) to 300% (mono dominance), and values of more than 10% for a species indicate a species is ecologically important [35].

**Fr**: Relative frequency (%) is obtained from the relationship between the number of individuals of species *i* (*ni*) and the total number of sampled individuals.

$${\text{Fr}}=\frac{\mathrm{Frequency\,of\,a\,species }({\text{Fi}})}{\mathrm{Total\,frequency\,of\,all\,species }({\text{F}})}\times100$$

$${\text{Fi}}=\frac{\mathrm{Number\,of\,recorded\,individus\,of\,a\,species }(ni)}{\mathrm{Total\,number\,of\,individuals\,of\,all\,species }(N)}\times 100$$

**Dr**: Relative density (%) is the percentage of the density of each sampled species (*Di*) and the total density of all species (*Dt*).

$${\text{Dr}}=\frac{\mathrm{Density\,of\,a\,species }(Di)}{\mathrm{Total\,density\,of\,all\,species }(D)}\times 100$$

$${\text{Di}}=\frac{\mathrm{Number\,of\,individus\,of\,species i}}{\mathrm{Total\,sampled\,area\,in\,hectare}}$$

**Gr**: Relative dominance (%) is the ratio between the total basal area of a species and the total basal area of all species

$${\text{Gr}}=\frac{\mathrm{Dominance\,of\, a\,species }({\text{Gri}})}{\mathrm{Total\,Dominance\,of\,all\,species }(D)}\times 100$$

$${\text{Gri}}=\frac{\mathrm{Total\,basal\,area\,of\,a\,species }({\text{Bai}})}{\mathrm{Total\,sampled\,area}}$$

$${\text{Basal}}\,{\text{area}} = \frac{{\pi \times \left( {{\text{DBH}}} \right)^{2} }}{4}$$

To assess, the effect of each factor on the plant use-availability dynamic (PUD), it was modelized as:

$${\text{PUD}}=\frac{{\text{UV}}}{\mathrm{Log }({\text{IVI}}+1)}$$

With IVI > 0, ie for available woody species.

The logarithmic transformation was used for availability data normalization.

The plant use-availability dynamic (PUD) was conceptualized as a function of plant use (UV) and availability (IVI). It includes the two parameters in a single mathematical function. Its variation indicated that of the correlation of the two parameters (UV & IVI).

Afterwards, regression models were used to highlight the significance of each parameter effect. The PUD was the response factor, while growth form (tree and shrub), phytodistrict, ethnicity, sex, age, instruction, marital status, household status, and main activity (Table 2), were the explanatory ones. The age distribution follows Ahoyo et al. [36]: Young (age < 40), Older (40 ≤ age < 60), and Elder (Age ≥ 60).

The phytodistricts effect on the PUD was assessed using a least significant difference (LSD) test. Concerning the social factors, the significance of their effects was screened through a linear mixed model. On the one hand, phytodistrict was considered as a fixed factor for ethnicity and age (random factors) effects assessment, and activity was the fixed factor for age ones, on the other hand. Some ethnics were grouped according to the similarity of their culture, to avoid the effect of underrepresentation. There are Fon, Goun and Adja (as Fon); Gourmache, Boo, Lokpa, Pila Pila, Wama, Biali and Yom (Gourmache); Peuhl and Gando (Peuhl); Xoli, Ayizo and Tori (Xoli); and Yoruba, Ifè and Isha (Yoruba).

To evaluate the effect of ecological diversity on ethnomedicinal knowledge, the Pearson correlation test was applied to diversity indices considering inventoried species in the survey plots and those which were cited by people. The diversity index was computed following Shannon [37]:

$$H=-\sum_{i=1}^{{\text{S}}}Pi {{\text{log}}}_{2}Pi$$

With *H*, the biodiversity index of Shannon; *i*, an inventoried species, S, the species richness; *Pi*, the species *i* relative frequency (*Pi* = *ni*/N), *ni* is the cited number of the species *i*, and *N* is the total cited number of all species.

The overall evaluation considered use and availability indices of species per phytodistrict as the relationship between usefulness and appearance might be specific to the area of influence [38].

All analyses were performed with R software, version 3.4.0 [39].

## Results

### Phytodistict and growth form effects on the plant use-availability dynamic (PUD)

A hundred and fifty-nine medicinal woody plants were sampled in total. Among them, were 103 trees and 56 shrubs (Additional file 1, Fig. 2). The applied regression showed a significant effect (*p* value: 0.0048) of growth form, and its interaction with phytodistrict on PUD variation (Table 3). Indeed, the growth form “tree” is responsible for the observed sensibility. The less available trees in the wild were the most used by people to take care of in general (Table 4). This observation is particularly accurate in the phytodistricts 3, 4, 8 and 10 (Table 5, Fig. 3). The most needful tree species of these phytodistricts were *Azadirachta indica*, *Cassia siberina*, *Cola millenii*, *Khaya senegalensis*, *Kigelia africana*, *Newbouldia laevis*, *Parkia biglobosa*, *Prosopis africana*, *Pseudrocedrela kotschyi*, *Pterocarpus erinaceus*, *Treculia africana*, *Uapaca heudelotii*, and *Vitelaria paradoxa* (Table 6). The tree species availability does not have any effect on their usage within the remaining phytodistricts [1, 2, 5–7, 9].

**Fig. 2:**
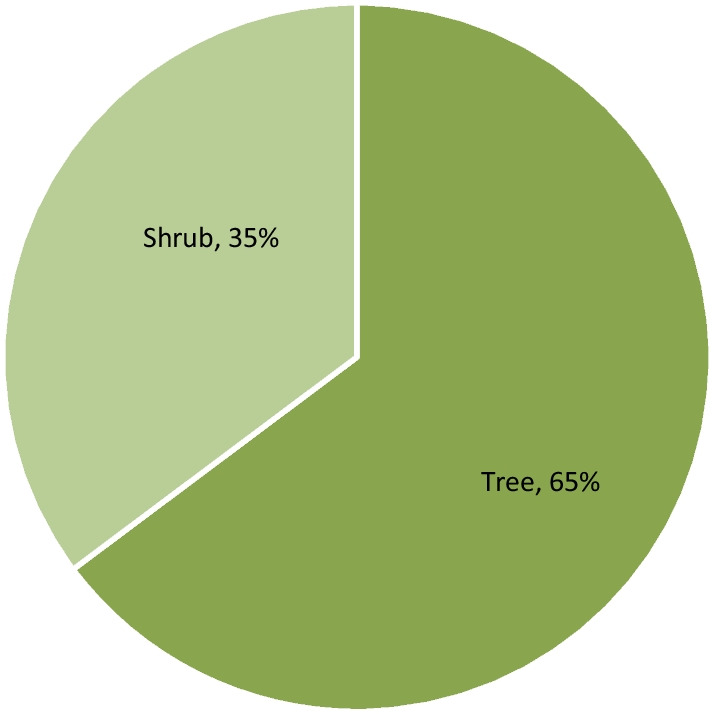
Life forms distribution among the sampled species

**Table 3:** Linear general Model of PUD sensibility to phytodistrict and life form (Terms with significant effects are in bold)

Variables	Estimation	Error	*t* value	*p* value
Intercept	0.089	0.013	6.639	41e−11***
Phytodistrict	0.077	0.040	1.909	0.058
**Life form**	**0.217**	**0.104**	**2.078**	0.039*
**Phytodistrict: Life form**	**− 0.033**	**0.014**	**− 2.322**	0.021*

Significativity codes: 0 ‘***’ 0.001 ‘**’ 0.01 ‘*’ 0.05 ‘.’ 0.1 ‘’

**Table 4:** Single growth form effect on PUD (Terms with significant effects are in bold)

Variables	Estimation	Error	*t* value	*p* value
Intercept (Shrub)	0.094	0.079	1.195	0.239
Intercept (Tree)	0.311	0.047	6.565	8.66e−10***
Shrub	0.011	0.011	1.019	0.314
**Tree**	**− 0.021**	**0.006**	**− 3.392**	0.0008***

Significativity codes: 0 ‘***’ 0.001 ‘**’ 0.01 ‘*’ 0.05 ‘.’ 0.1 ‘’

**Table 5:** LSD test applied for the phytodistrict effect on PUD considering the tree species only (Terms with significant effects are in bold)

Phytodistrocts	EAH	std	*r*	LCL	UCL	Min	Max
1	0.524	0.642	5	0.253	0.794	0.08	1.60
2	0.590	0.331	5	0.319	0.860	0.23	0.98
**3**	**0.156**	**0.204**	**5**	**− 0.114**	**0.426**	**0.02**	**0.50**
**4**	**0.110**	**0.126**	**5**	**− 0.160**	**0.380**	**0.03**	**0.33**
5	0.278	0.320	5	0.007	0.548	0.03	0.80
6	0.390	0.120	5	0.119	0.660	0.22	0.56
7	0.274	0.176	5	0.003	0.544	0.14	0.54
**8**	**0.212**	**0.218**	**5**	**− 0.058**	**0.482**	**0.10**	**0.60**
9	0.492	0.150	5	0.221	0.762	0.35	0.69
**10**	**0.170**	**0.041**	**5**	**− 0.100**	**0.440**	**0.13**	**0.22**

**Fig. 3:**
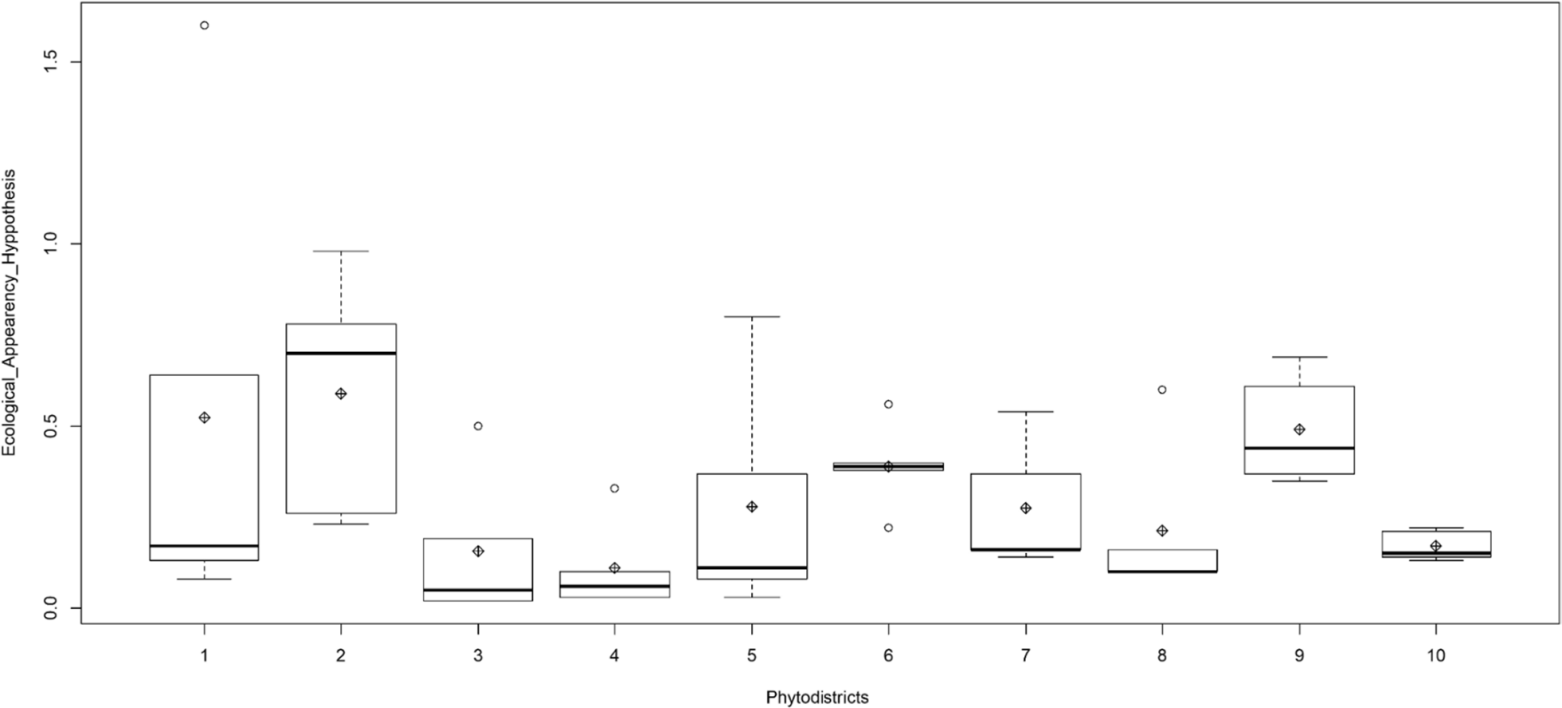
PUD sensibility following phitodistrics, considering tree species. 1. Coastal, 2. Pobe, 3. Ouemey valley, 4 Plateau, 5. Zou, 6. Bassila, 7. South borgou, 8. North borgou, 9. Atacora chain, 10. Mekrou-Pendjari

**Table 6:** Most used tree species PUD parameters in the phytodistricts 3, 4, 8, and 10

Families	Species	UV	IVI (%)
Anacardiaceae	*Lannea acida* A. Rich	0.24	9.47
Anacardiaceae	*Lannea barteri* (Olïv.) Engl	0.30	13.80
Bignoniaceae	*Kigelia africana* (Lam.) Benth	0.83	0.00
Bignoniaceae	*Newbouldia laevis* (P. Beauv.) Seem	0.35	0.00
Combretaceae	*Terminalia leiocarpa (DC.) Baill*	0.25	26.75
Combretaceae	*Combretum collinum* Fresen	0.21	22.73
Fabaceae	*Afzelia africana* Pers	0.50	30.95
Fabaceae	*Burkea africana* Hook	0.19	15.19
Fabaceae	*Cassia sieberiana* DC	0.40	0.00
Fabaceae	*Daniellia oliveri* (Rolfe) Hutch. & Dalziel	0.34	12.68
Fabaceae	*Detarium microcarpum* Guill. & Perr	0.16	10.70
Fabaceae	*Parkia biglobosa* (Jacq.) G. Don	0.54	0.00
Fabaceae	*Prosopis Africana* (GuilI. & Perr.) Taub	0.41	0.00
Fabaceae	*Pterocarpus erinaceus* Poir	0.46	0.00
Malvaceae	*Cola millenii* K. Schum	0.29	0.00
Meliaceae	*Azadirachta indica* A. Juss	0.40	0.00
Meliaceae	*Khaya senegalensis* (Desv.) A. Juss	2.28	0.00
Meliaceae	*Pseudrocedrela kotschyi* (Schweinf.) Harms	0.30	0.00
Moraceae	*Ficus sur* Forssk	0.06	12.82
Moraceae	*Treculia africana* Decne. Ex Trecul	0.29	0.00
Ochnaceae	*Lophira lanceolata* Tiegh. ex Keay	0.31	0.87
Phyllanthaceae	*Uapaca heudelotii* Baill	0.16	0.00
Sapotaceae	*Vitellaria paradoxa* C. F. Gaertn	0.79	0.00

UV: Sum of tree Use Value in the mentioned phytodistricts, IVI: Sum of tree Importance value index in the mentioned phytodistricts

### Socio-demography effects on the plant use-availability dynamic (PUD)

Plant use-availability dynamic varies significantly according to social factors (*p* value: 0.007). Ethnicity, age and main activity were the most quoted social factors which influenced the PUD sensibility. Ethnicity and age have various effects, considering the phytodistricts. Moreover, age influence changes following the main activity (Tables 7 and 8).

**Table 7:** PUD sensibility to phytodistrict and socio-demographic factors

Variables	Estimation	Error	t value	Pr ( >|t|)
Intercept	0.159	0.352	0.453	0.65
Phytodistrict	6.3e−03	0.003	− 0.206	0.83
Sex	0.003	0.226	− 0.173	0.86
Ethnicity	− 5.07e−03	0.001	− 0.298	0.76
Age	− 1.82e−03	3.6e−03	− 0.494	0.62
Instruction	− 4.95e−02	1.53e−01	− 0.324	0.74
Marital status	6.93e−02	1.18e−01	0.587	0.55
Household status	7.40e−02	2.19e−01	0.337	0.73
Activity	− 5.304e−02	5.10e−02	− 1.039	0.29
Phytodistrict: Sex	1.308e−02	1.61e−02	0.808	0.41
Phytodistrict: Ethnicity	1.839e−03	8.43e−04	2.182	0.03*
Phytodistrict: Age	− 3.71e−04	1.46e−04	− 2.537	0.01*
Phytodistrict: Instruction	− 1.54e−03	2.94e−03	− 0.525	0.59
Phytodistrict: Marital status	1.7e−03	5.11e−03	0.351	0.72
Phytodistrict: Household status	− 5.46e−03	2.08e−02	− 0.263	0.79
Phytodistrict: Activity	− 9.79e−04	1.80e−03	− 0.543	0.58
Sex: Ethnicity	− 3.71e−03	9.10e−03	− 0.408	0.68
Sex: Age	1.23e−03	1.87e−03	0.658	0.51
Sex:instruction	1.05e−02	5.79e−02	0.183	0.85
Sex: Marital status	− 2.10e−02	3.90e−02	− 0.540	0.58
Sex: Household status	6.47e−03	8.08e−02	0.080	0.93
Sex: Activity	− 9.201e−03	2.92e−02	− 0.314	0.75
Ethnicity: Age	− 1.931e−05	1.26e−04	− 0.153	0.87
Ethnicity:instruction	4.954e−03	3.06e−03	1.616	0.10
Ethnicity: Marital status	− 3.443e−03	3.84e−03	− 0.894	0.37
Ethnicity: Household status	1.928e−03	4.24e−03	0.454	0.64
Ethnicity: Activity	− 2.895e−04	1.54e−03	− 0.188	0.85
Age: Instruction	− 6.933e−04	6.79e−04	− 1.020	0.30
Age: Marital status	4.573e−04	7.40e−04	0.617	0.53
Age: Household status	− 9.352e−04	2.49e−03	− 0.375	0.70
Age: Activity	1.116e−03	2.89e−04	3.861	0.00***
Instruction: Marital status	− 9.77e−04	1.48e−02	− 0.066	0.94
Instruction: Household status	5.439e−02	1.22e−01	0.443	0.65
Instruction: Activity	1.844e−03	6.12e−03	0.301	0.76
Marital status: Household status	− 4.3e−02	8.20e−02	− 0.524	0.60
Marital status: Activity	− 8.403e−03	7.77e−03	− 1.081	0.28
Household status: Activity	1.980e−02	3.15e−02	0.627	0.53

Significativity codes: 0 ‘***’ 0.001 ‘**’ 0.01 ‘*’ 0.05 ‘.’ 0.1 ‘’ 1

**Table 8:** PUD sensibility to people age (Ls means test)

AGE	Mean	SE	*df*	LCL	UCL
Olders	0.076	0.035	2.49	− 0.052	0.204
Youngs	0.130	0.077	0.72	− 2.461	2.722
Elders	0.060	0.008	0.54	− 0.894	1.015

**Fig. 4:**
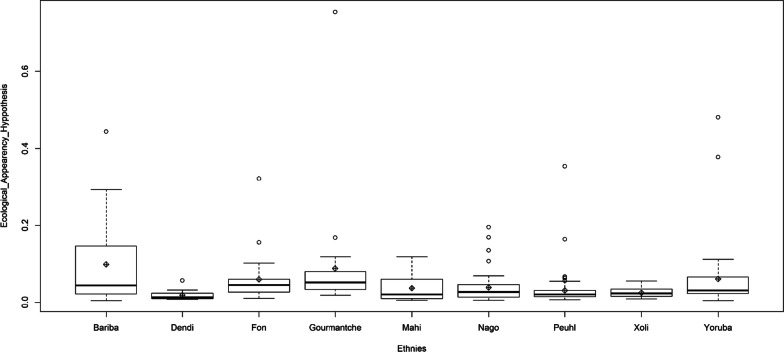
Plant use-availability dynamic following ethnicity through phytodistrics

Peuhl, Nago, Fon, Ifè, Bariba Mahi and Xoli ethnics, mostly use the most available species. On the contrary, Gourma, Dendi, Isha, Yom, Yoruba, Boo, Lokpa, Adja, Pila Pila, Ayizo, Goun, Tori, Wama, Biali, Gando people use more the less available species (Fig. 4).

**Fig. 5:**
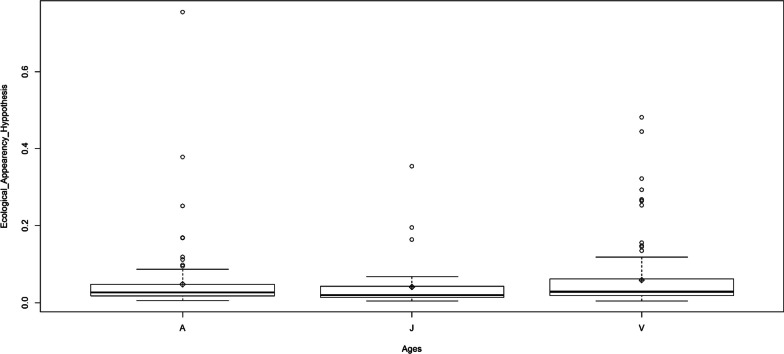
Plant use-availability dynamic following age through phitodistrics. A, Adult; J, Young; V, Old

**Fig. 6:**
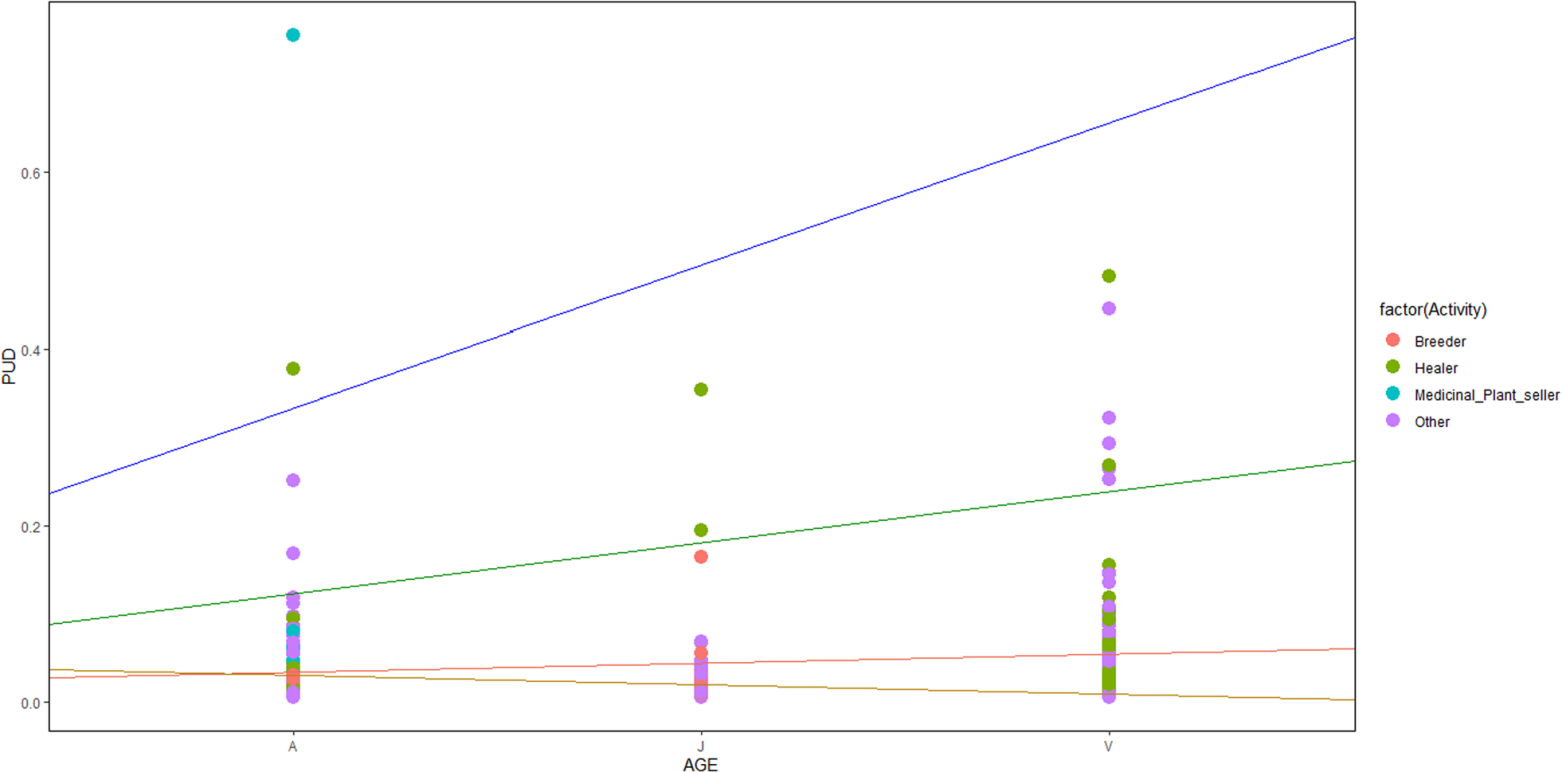
Plant Use-availability following age over main activity. A, Adult; J, Young; V, Old

Concerning the age factor, young people mostly use the available species, contrary to their elders, who mostly use the less available ones (Fig. 5). Indeed, among the medicinal plant sellers, traditional healers, and breeders, people of all age classes seem to mostly use the less available tree species. The contrary trend was observed concerning the other plant professionals (Fig. 6).

### Wild ecological diversity effect on ethnomedicinal knowledge diversity

The existent plant diversity in the surrounding environment seems to have no effect on plant selection for medicinal purposes (r: − 0.293; *p* value: 0.403). Indeed, the most diversified environment does not necessarily shelter the most knowledgeable people, considering traditional medicine practice.

## Discussion

### Plant use-availability dynamic (PUD) sensitivity to growth form

Growth form and its interaction with phytodistrict have a significant effect on PUD sensitivity. According to the availability hypothesis, the most available species will be the most used [21]. The contrary of this original assertion was found. Indeed, the less available trees were the most used in the phytodistricts 3 (Plateau), 4 (Oueme Valley), 8 (North-Borgou) and 10 (Mekrou-Pendjari). Such contradictions in natural resource use were already raised by the maladaptation theory [40]. Indeed, evolutionary biologists tend to emphasize the power of natural selection in generating adaptation to local environments [41–43]. Thus, a given environment may hold the most adapted species, both humans and plants. People of phytodistricts 3, 4, 8 and 10 appear maladapted from an evolutionary perspective [40]. They must adapt to specific environmental conditions, which includes learning to use the most available plants [44] for them.

In these areas, where tree seems to be the predominant growth form (versus shrub), the observed maladaptation could follow invasion dynamics and their responses to environmental change [45]. The invasive species used by people may be common after an adaptation period. This maladaptation could have a hereditary basis. Hence, people still use the species, they learn from their parents, even if they become scarce. Furthermore, this maladaptation trend could be corrected by targeted reforestation, increasing the most wanted species availability [46]. As suggested by Phillips and Gentry [12], the communities themselves could favour the abundance of desired species.

On the other hand, this lack of correlation could be due to the overexploitation of most useful tree species through destructive harvesting causing their scarcity.

The shrub species were used without availability consideration. The use of a shrub species depends, thus on its ethnobotanical importance [47], rather than its availability. Moreover, tree species have been found more useful than shrubs [48]. The low availability of tree species could be due to their involvement in some other use categories like technology. On the other hand, traditional medicinal knowledge being hereditary [49], same species were used through several generations. Thus, despite the decrease in some native tree species’ availability, they continue to be solicited by people.

Whether plant selection follows species availability in general, this trend is not accurate considering medicinal uses. The findings are close to those of de Lucena et al. [44], who confirmed the availability hypothesis for energy and construction purposes, rather for medicinal ones. In the same frame, Lawrence et al. [50] suggest that availability is more closely related to ecological dominance measurements in the category of wood used for construction. Sometimes, a strong confirmation of the PUD was found when, the individual’s components of the species availability index were taken into account, even, for medicinal purposes. The species frequency of citation, for example, had a good correlation with their use value within medicinal category [44, 50].

### People’s characteristic effects on plant selection considering their availability

In ethnobotany, several studies were devoted to highlighting social factors, which determine plant selection. Some of the PUD components were sometimes assessed at this end [20]. For traditional medicinal knowledge in Benin, PUD varies significantly according to social factors. Ethnicity, age and main activity were the most quoted social factors which influenced the PUD. Ethnicity and age have various effects, considering the phytodistricts. Moreover, age’s influence changes following the main activity. Generally, cultural factors determine the nature of species use, and the most abundant species are not always the most important [44, 51].

The effects of age and ethnicity on plant selection were revealed by several ethnobotanical studies [22, 26, 52]. Traditional medicinal knowledge reached the greatest importance with elderly people [52]. They often hold useful knowledge on some scarce species.

Former studies [44] showed the sex effect in the PUD modulation. However, these studies involved several use categories whose species use follows sex distribution. Sex does not lead to species usage in the medicinal field. Furthermore, although men generally possess more knowledge concerning wood products, in contrast to women, who tend to know more about non-woody [53], the misrepresentation of women in the present study, could inhibit the sex effect on the PUD. The observed non-significant effect of sex could also be caused that sometimes; men and women shared similar knowledge about the use of medicinal plants [54].

### Ethnomedicinal knowledge and species diversity

Although a strong correlation between areas of high biodiversity and cultural diversity has been found at a global scale [55–57], plant selection for medicinal purposes in Benin follows other criteria than the surrounding environment diversity. Thus, the inhabitants of a well-ecologically diversified area do not necessarily have a diversified knowledge of the species use, as found by Lucena et al. [44]. The diversity pattern that has been observed in the wild, contrasted with species selection [58–60]. The weak correlation between wild diversity and that of ethno medicinal knowledge could be also explained by the environmental scarcity compensation phenomenon effect [61, 62]. Indeed, the highest diversity of ethnomedicinal knowledge was often recorded among inhabitants of the least diversified environment and vice versa. Although the surveys were not undertaken in home gardens, people, sometimes, manage the scarce medicinal plants in their home gardens whether they do not have easy access to them in the surrounding environment [62, 63]. Thus, the mostly used tree species which have lesser availability in wild could be more available in home gardens where they are readily available for use.

In general, people still use the inherited species, even, if they become scarce. They go often very far to their home for known useful species gathering. Even if some species are abundantly available for potential use, actual use can still be constrained by species-specific taboos [64]. Moreover, Species substitution for equivalent medicinal effects was seem not yet integrated into the people’s habit.

Furthermore, the weak availability of the most useful medicinal species could be due to their involvement within other use categories. On the contrary, the less used species may have a high availability due to their weak solicitation [65]. On the other hand, the PUD test is inferred from the availability and optimal foraging hypothesis, which originally involved herbivory susceptibility to plant species that are visible and abundant [66]. The theory predicts that foraging organisms will balance the benefit received from food with the effort it took to search for and eat that food. From the point of view of its hypothesis, people tend to use the most available and affordable resources.

Unlike free grazing in herbivory, local communities design cultural institutions and social norms to regulate access and to sanction appropriate corrective measures when contravention to the governing rules of common resources is detected [51, 67]. Thus, all visible plant species do not gather for use, especially, for medicinal ones.

The complexity of the overall ecological model’s explanation and/or understanding induced the assertion that “All ecological models are wrong, but some are useful” [67]. The plant use-availability dynamic one is useful for better management of biodiversity.

Some limitations of this study deserve to be acknowledged.

The first concerns the considered life form. The distinction between trees and shrubs is mainly based on plant height and this is a possible source of many ambiguities. Indeed, despite the uniform frame for the definition of trees and shrubs provided in Europe [68], there is still an ambiguous boundary between these two concepts The morphology of a species depends on stational parameters, such as latitude, altitude, exposure, soil condition [68], temperature, humidity, rainfall, etc. The height is overall a constant parameter for making distinctions between trees and shrubs.

Secondly, the variation of the plant use-availability dynamic (PUD) is based on adult individuals only. As the luck of seedlings to reach adult stand is ensured, the study used stable data, eg: Diameter at Breast Height (DBH) for adult individuals.

## Conclusion

The plant use-availability dynamic (PUD) was among the well-discussed theories in ethnobotany studies, through different names. The study presents a new approach to assess its sensitivity, by a synergic use of the plant use value, and the age, gender, and dynamics of knowledge hypothesis. It appears that plant use for medicinal purposes is not related to only available resources abundance, but also to people’s social factors.

In ethno-medicine, the PUD is sensitive to ethnicity, sex, growth form, and phytodistrict. Native tree species, although scarce, seem more useful than shrubs. Moreover, a well-ecological diversified area does not imply a highly diversified ethno-medicinal knowledge. Thus, the ecological resource use behaviour of local people is far more complex than an exclusive association with an abundance of ecological resources.

Home gardens which remain a good management area for the lesser available species in wild, should be promoted or reinforced in some regions. To prevent the erosion of biodiversity, and allow seed keeping, some phytodistricts of Benin, especially those of Plateau, Oueme Valley, North-Borgou and Mekrou-Pendjari, need to be reforested. *Azadirachta indica*, *Cassia siberina*, *Cola milenii*, *Khaya senegalensis*, *Kigelia africana*, *Newbouldia laevis*, *Parkia biglobosa*, *Prosopis africana*, *Pseudrocedrela kotschyi*, *Pterocarpus erinaceus*, *Treculia africana*, *Uapaca heudelotii*, and *Vitelaria paradoxa* should be prioritized for in situ and ex situ conservation plans.

Since the simple species availability does not explain their medicinal use, participative biodiversity management which recognises the necessary local people involvement, may be strengthened for a better protection of vulnerable species or ecosystems. Indeed, traditional society which applies dynamic and adaptive socio-ecological knowledge systems (beliefs, knowledge, and practices) as an integral part of managing the delicate balance of “use-protection” regimes may be reinforced.

### Supplementary Information

**Additional file 1. Table A1.** Total sampled species per phytodistrict.

## Data Availability

The original contributions presented in the study are included in the article. Further inquiries can be directed to the corresponding author.

## Abbreviations

UV: Use value
IVI: Importance value indices
PUD: Use-availability dynamic
H: Biodiversity index of Shannon

## References

[1] Haq SM, Pieroni A, Bussmann RW, ElGawad AMA, El-Ansary HO (2023). Integrating traditional ecological knowledge into habitat restoration: implications for meeting forest restoration challenges. J Ethnobiol Ethnomed.

[2] Adjahossou SGC, Houéhanou DT, Toyi M, Salako VK, Ahoyo CC, Lesse P, Tente B, Houinato MRB (2019). Dépendance socioculturelle des connaissances locales des usages de Isoberlinia spp. au Moyen-Bénin, Afrique de l’Ouest. Bois et Forêts des Tropiques.

[3] Robinson MM, Zhang X (2011). The world medicines situation, traditional medicines: Global situation, issues and challenges.

[4] Guo Z, Zhang L, Li Y (2010). Increased dependence of humans on ecosystem services and biodiversity. PLoS ONE.

[5] Lucena RFP, Medeiros PM, Lima Araújo E, Alves AGC, Albuquerque UP (2012). The ecological apparency hypothesis and the importance of useful plants in rural communities from Northeastern Brazil: an assessment based on use value. J Environ Manag.

[6] Gaoue OG, Coe MA, Bond M, Hart G, Seyler BC, McMillen H (2017). Theories and major hypotheses in ethnobotany. Econ Bot.

[7] Albuquerque UP (2006). Re-examining hypotheses concerning the use and knowledge of medicinal plants a study in the Caatinga vegetation of NE Brazil. J Ethnobiol Ethnomed.

[8] Voeks RA (2004). Disturbance pharmacopoeias: medicine and myth from the humid tropics. Ann Assoc Am Geogr.

[9] Estomba D, Ladio A, Lozada M (2006). Medicinal wild plant knowledge and gathering patterns in a Mapuche community from Northwestern Patagonia. J Ethnopharmacol.

[10] Low BS (1996). Behavioral ecology of conservation in traditional societies. Hum Nat.

[11] Van de Waal DB, Elser JJ, Martiny AC, Sterner RW, Cotner JB (2018). Editorial: progress in ecological stoichiometry. Front Microbiol.

[12] Phillips O, Gentry AH (1993). The useful plants of Tambopata, Peru: II. Additional hypothesis testing in quantitative ethnobotany. Econ Bot.

[13] Phillips JC (1996). Stretched exponential relaxation in molecular and electronic glasses. Rep Prog Phys.

[14] da Silva Filho AA, Albuquerque S, Silva ML, Eberlin MN, Tomazela DM, Bastos JK (2004). Tetrahydrofuran lignans from Nectandra egapotamica with trypanocidal activity. J Nat Prod.

[15] Lawrence D (2005). Biomass accumulation after 10–200 years of shifting cultivation in Bornean rain forest. Ecology.

[16] Cunha WR, Crevelin EJ, Arantes GM, Crotti AEM, Silva MLA, Furtado NAC, Albuquerque S, Ferreira DS (2006). A study of the trypanocidal activity of triterpene acids isolated from Miconia species. Phytotherapia Resources Int J.

[17] Lucena MI, Andrade RJ, Martínez C, Ulzurrun E, García-Martín E, Borraz Y, Fernández MC, Romero-Gomez M, Castiella A, Planas R (2008). Glutathione S-transferase m1 and t1 null genotypes increase susceptibility to idiosyncratic drug-induced liver injury. Hepatology.

[18] Watson A, Alessa L, Glaspell B (2003). The relationship between traditional ecological knowledge, evolving cultures, and wilderness protection in the circumpolar north. Conserv Ecol.

[19] Pautasso M (2007). Scale dependence of the correlation between human population presence and vertebrate and plant species richness. Ecological Letters.

[20] Gonçalves JT, Schafer ST, Gage FH (2016). Adult neurogenesis in the hippocampus: from stem cells to behavior. Cell.

[21] Albuquerque UP, Lucena RFP (2005). Can apparency affect the use of plants by local people in tropical forests?. Interciencia.

[22] Ahoyo CC, Houehanou TD, Yaoitcha AS, Prinz K, Assogbadjo AE, Adjahossou CSG, Hellwig F, Houinato MRB (2018). A quantitative ethnobotanical approach toward biodiversity conservation of useful woody species in Wari-Maro forest reserve (Benin, West Africa). Environ Dev Sustain.

[23] Gandji K, Salako VK, Fandohan B, Assogbadjo AE, Glèlè Kakaï RL (2018). Factors determining the use and cultivation of *Moringa oleifera* Lam. in the Republic of Benin. Econ Bot.

[24] Soares DT, Sfair JC, Reyes-García V, Baldauf C (2017). Plant knowledge and current uses of woody flora in three cultural groups of the Brazilian semiarid region: does culture matter?. Econ Bot.

[25] Cakpo YT, Tovissodé C, Biaou C, Toko I, Lougbégnon T, Sinsin B, Korb J (2017). Ethnobotanic assessment of debarked medicinal plants in southern Benin: the case of Lokoli swampy forest and Lama protected forest. Int J Agric Environ Res.

[26] McCarter J, Gavin MC (2015). Assessing variation and diversity of ethnomedical knowledge: a case study from Malekula Island, Vanuatu. Econ Bot.

[27] Hanazaki N, Herbst DF, Marques MS, Vandebroek I (2013). Evidence of the shifting baseline syndrome in ethnobotanical research. J Ethnobiol Ethnomed.

[28] Souto T, Ticktin T (2012). Understanding interrelationships among predictors (age, gender, and origin) of local ecological knowledge. Econ Bot.

[29] Adomou AC (2005) Plant Vegetation patterns and environmental gradients in Benin: implications for biogeography and conservation. Ph.D. thesis Wageningen University, Wageningen, p 133

[30] Adomou C, Agbani OP, Sinsin B. Phytogéographie du Bénin. In: Neuenschwander P, Sinsin B, Goergen G (eds) Protection de la Nature en Afrique de l’Ouest: Une Liste Rouge pour le Bénin. Nature Conservation in West Africa: Red List for Benin, Ed. International Institute of Tropical Agriculture, Nigeria, pp. 14–20; 2011.

[31] Faihun AML, Akouedegni CG, Olounlade PA, Adenile DA, Hounzangbe-Adote SM (2017). Typologie des élevages de cobayes (*Cavia porcellus*) au Bénin. Int J Biol Chem Sci.

[32] Akoègninou A, Van der Burg WJ, Van der Maesen LJG (2006). Flore analytique du Bénin.

[33] ISE. The ISE (International Society of Ethnobiology) Code of Ethics (2008). http://www.ethnobiology.net/what-we-do/core-programs/ise-ethics-program/code-of-ethics/

[34] Rossato SC, Leitao FH, Begossi A (1999). Ethnobotany of caiçaras of the Atlantic Forest coast (Brazil). Econ Bot.

[35] Reitsma JM. Forest vegetation in Gabon. Tropenbos Technical Series 1. The Netherlands, p. 142; 1988.

[36] Ahoyo CC, Houehanou TD, Yaoitcha AS, Prinz K, Glèlè Kakaï RL, Sinsin BA, Houinato MRB (2021). Traditional medicinal knowledge of woody species across climatic zones in Benin (West Africa). J Ethnopharmacol.

[37] Shannon CE, Weaver W (1963). The mathematical theory of communication.

[38] Guèze M, Luz AC, Paneque-Gálvez J, Macía MJ, Orta-Martínez M, Pino J, Reyes-García V (2014). Are ecologically important tree species the most useful? A case study from indigenous people in the Bolivian Amazon. Econ Bot.

[39] R Core Team R. A language and environment for statistical computing. R Foundation for Statistical Computing, Vienna; 2017. https://www.R-project.org/.

[40] Brady SP, Bolnick DI, Barrett RDH, Chapman L, Crispo E, Derry AM, Eckert CG, Fraser DJ, Fussmann GF, Gonzalez A, Guichard F, Lamy T, Lane J, McAdam AG, Newman AEM, Paccard A, Robertson B, Rolshausen G, Schulte PM, Simons AM, Vellend M, Hendry A (2019). Understanding maladaptation by uniting ecological and evolutionary perspectives. Am Nat.

[41] Darwin C (1859). On the origin of species by Means of Natural Selection.

[42] Bell G (2008). Selection: the mechanism of evolution.

[43] Hendry AP (2016). Eco-evolutionary dynamics.

[44] Lucena RFP, de Lima Araújo E, de Albuquerque UP (2007). Does the local availability of woody caatinga plants (Northeastern Brazil) explain their use value?. Econ Bot.

[45] Urban MC, Scarpa A, Travis JMJ, Bocedi G (2019). Maladapted prey subsidize predators and facilitate range expansion. Am Nat.

[46] Cotto O, Sandell L, Chevin LM, Ronce O (2019). Maladaptive shifts in life history in a changing environment. Am Nat.

[47] Diedhiou A, Probst JC, Hardin JW, Martin AB, Xirasagar S (2010). Relationship between presence of a reported medical home and emergency department use among children with asthma. Med Care Resources Revue.

[48] Dassou HG, Ogni CA, Yedomonhan H, Adomou AC, Tossou M, Dougnon JT, Akoègninou A (2014). Diversité, usages vétérinaires et vulnérabilité des plantes médicinales au Nord-Bénin. Int J Biol Chem Sci.

[49] Laurendeau G. 2011. Usages des plantes par les Pekuakamiulnuatsh: étude sur la transmission des savoirs dans la communauté ilnu de Mashteuiatsh. Mémoire de maitrise, Université Laval. http://hdl.handle.net/20.500.11794/22815.

[50] Lawrence A, Phillips OL, Reategui A, Lopez M, Rose S, Wood D, Farfan AJ (2005). Local values for harvested forest plants in Madre de Dios, Peru: towards a more contextualised interpretation of quantitative ethnobotanical data. Biodivers Conserv.

[51] Mulugheta GA, Paxie WC (2019). Revealing the predominance of culture over the ecological abundance of resources in shaping local people’s forest and tree species use behavior: the case of the Vhavenda people. S Afr Sustain.

[52] Houehanou TD, Assogbadjo AE, Glèlè Kakaï R, Houinato M, Sinsin B (2011). Valuation of local preferred uses and traditional ecological knowledge in relation to three multipurpose tree species in Benin (West Africa). For Policy Econ.

[53] Taita P (2003). Use of woody plants by locals in mare aux hippopotames biosphere reserve in Western Burkina Faso. Biodivers Conserv.

[54] Matavele J, Habib M (2000). Ethnobotany in Cabo Delgado, mozambique: use of medicinal plants. Environ Dev Sustain.

[55] Pretty J, Adams B, Berkes F, de Athayde SF, Dudley N, Hunn E, Maffi L, Milton K, Rapport D, Robbins P, Sterling E, Stolton S, Tsing A, Vintinner E, Pilgrim S (2009). The intersections of biological diversity and cultural diversity: towards integration. Conserv Soc.

[56] Gorenflo LJ, Romaine S, Mittermeier RA, Walker-Painemilla K (2012). Cooccurrence of linguistic and biological diversity in biodiversity hotspots and high biodiversity wilderness areas. Proc Natl Acad Sci.

[57] Fagúndez J, Izco J (2016). Diversity patterns of plant place names reveal connections with environmental and social factors. Appl Geogr.

[58] Galeano G (2000). Forest use at the pacific coast of Chocó, Colombia: a quantitative approach. Econ Bot.

[59] Cunha LVF, Albuquerque UP (2006). Quantitative ethnobotany in an atlantic forest fragment of Northeastern Brazil-implications to conservation. Environ Monit Assess.

[60] Ferraz JSF, Meunier IMJ, Albuquerque UP (2005). Conhecimento sobre espécies lenhosas úteis da mata ciliar do Riacho do Navio, Floresta, Pernambuco. Zonas Áridas.

[61] Larios C, Casas A, Vallejo M, Moreno-Calles AI, Blancas J (2013). Plant management and biodiversity conservation in Náhuatl homegardens of the Tehuacán Valley, Mexico. J Ethnobiol Ethnomed.

[62] Kujawska M, Zamudio F, Montti L, Carrillo VP (2018). Effects of landscape structure on medicinal plant richness in home gardens: evidence for the environmental scarcity compensation hypothesis. Econ Bot.

[63] Poot-Pool WS, van der Wal H, Flores-Guido S, Pat-Fernández JM, Esparza-Olguín L (2015). Home garden agrobiodiversity differentiates along a rural—peri-urban gradient in Campeche, Mexico. Econ Bot.

[64] Aswani S, Lemahieu A, Sauer WH (2018). Global trends of local ecological knowledge and future implications. PLoS ONE.

[65] Soldati GT, de Medeiros PM, Duque-Brasil R, Coelho FMG, Albuquerque UP (2017). How do people select plants for use? Matching the ecological apparency hypothesis with optimal foraging theory. Environ Dev Sustain.

[66] Albuquerque UP, Medeiros PM, Almeida AL, Monteiro JM, Lins Neto EMF, de Melo JG, Santos JP (2007). Medicinal plants of the caatinga (semi-arid) vegetation of NE Brazil: a quantitative approach. J Ethnopharmacol.

[67] Ruiz-Mallén I, Corbera E (2013). Community-based conservation and traditional ecological knowledge: Implications for social-ecological resilience. Ecol Soc.

[68] Gschwantner T, Schadauer K, Vidal C, Lanz A, Tomppo E, di Cosmo L, Robert N, Englert Duursma D, Lawrence M (2009). Common tree definitions for national forest inventories in Europe. Silva Fennica.

